# Centers for Disease Control and Prevention Public Health Response to Humanitarian Emergencies, 2007–2016

**DOI:** 10.3201/eid2313.170473

**Published:** 2017-12

**Authors:** Andrew T. Boyd, Susan T. Cookson, Mark Anderson, Oleg O. Bilukha, Muireann Brennan, Thomas Handzel, Colleen Hardy, Farah Husain, Barbara Lopes Cardozo, Carlos Navarro Colorado, Cyrus Shahpar, Leisel Talley, Michael Toole, Michael Gerber

**Affiliations:** Centers for Disease Control and Prevention Epidemic Intelligence Service, Atlanta, Georgia, USA (A.T. Boyd);; Centers for Disease Control and Prevention, Atlanta (S.T. Cookson, M. Anderson, O.O. Bilukha, M. Brennan, T. Handzel, C. Hardy, F. Husain, B.L. Cardozo, C.N. Colorado, C. Shahpar, L. Talley, M. Gerber);; Burnet Institute, Melbourne, Victoria, Australia (M. Toole)

**Keywords:** humanitarian emergencies, complex emergencies, refugees, population displacement, Centers for Disease Control and Prevention, public health, epidemiology, emergency response, global health security

## Abstract

Humanitarian emergencies, including complex emergencies associated with fragile states or areas of conflict, affect millions of persons worldwide. Such emergencies threaten global health security and have complicated but predictable effects on public health. The Centers for Disease Control and Prevention (CDC) Emergency Response and Recovery Branch (ERRB) (Division of Global Health Protection, Center for Global Health) contributes to public health emergency responses by providing epidemiologic support for humanitarian health interventions. To capture the extent of this emergency response work for the past decade, we conducted a retrospective review of ERRB’s responses during 2007–2016. Responses were conducted across the world and in collaboration with national and international partners. Lessons from this work include the need to develop epidemiologic tools for use in resource-limited contexts, build local capacity for response and health systems recovery, and adapt responses to changing public health threats in fragile states. Through ERRB’s multisector expertise and ability to respond quickly, CDC guides humanitarian response to protect emergency-affected populations.

The number of persons affected by humanitarian emergencies worldwide is unprecedented; in 2016, the United Nations Office for the Coordination of Humanitarian Affairs estimated that 125 million persons needed humanitarian assistance (*1*). More than half of these, 65.3 million persons, have been forcibly displaced as a result of armed conflict, civil strife, or human rights violations. The number displaced has increased by 75% during the past 20 years and by 50% in just the past 5 years (*2*). Among these are 21.3 million refugees and 40.8 million internally displaced persons (IDPs) (*2*). Displaced persons might settle in temporary shelters or camps in resource-limited or politically unstable areas, straining local capacity to provide services. The effects of humanitarian emergencies can be exacerbated by political instability and weak governance associated with fragile states or areas of conflict (*3*), and this instability directly undermines global health security. In such unstable settings, the humanitarian community calls these crises complex emergencies (CEs) (Table) (*4*).

**Table:** Definitions of terms related to disasters and humanitarian emergencies

Term	Definition
Disaster	A serious disruption of the functioning of a community or a society involving widespread human, material, economic, or environmental losses and impacts that exceeds the ability of the affected community or society to cope using its own resources
Natural disaster	A disaster brought about by natural hazards
Human-made disaster	A disaster brought about by human activities or events (*4*)
Humanitarian emergency	A disaster resulting in the need for international support (humanitarian assistance) to meet the basic needs of the affected population (*4*)
Complex emergency	A humanitarian emergency associated with fragile states or areas of conflict, in which a total or considerable breakdown of authority has occurred (*4*)
Global health security	A state of collective protection of health through ensuring all countries can effectively prevent, detect, and respond to public health emergencies (*5*)

Although the underlying causes of humanitarian emergencies and CEs specifically are highly varied, the population displacement and health systems destabilization associated with emergencies have predictable public health consequences. A hallmark of CEs is increased mortality rates, sometimes >10-fold above baseline rates (*3*,*6*,*7*). Historically, the cause of the high morbidity and mortality rates have been infectious disease outbreaks; exacerbation of endemic infectious diseases; and acute malnutrition, often in high-density settlements with inadequate water, sanitation, shelter, and access to food (*3*,*7*–*10*). Increased availability of interventions for these conditions, coupled with a rise in conflicts in higher-income countries, have led to an increasing burden from chronic conditions such as tuberculosis, cardiovascular disease, and diabetes (*3*,*8*,*9*). Conflict-affected populations also have an elevated risk for injury from violence, including sexual and gender-based violence, and mental health conditions are common (*3*,*9*). Most displaced persons live in host communities, rather than in separate camps, contributing to poor or uncoordinated access to healthcare services (*9*). This inconsistent access continues to be problematic in protracted emergencies, during which public health services might be strained for years. Responding to the wide-ranging public health effects of CEs requires expertise in diverse sectors, such as vaccine-preventable and other infectious diseases; water, sanitation, and hygiene (WASH); nutrition; noncommunicable diseases; injury; sexual and reproductive health; and mental health. Equally varied are the epidemiologic approaches needed to effectively respond to CEs, including development of novel epidemiologic methods, rapid needs assessments, surveillance implementation and evaluation, outbreak investigations, and capacity building, often in resource-restricted and insecure environments.

The Centers for Disease Control and Prevention (CDC) has long been a leader in developing and understanding the epidemiology and public health effects of humanitarian emergencies and CEs specifically. CDC’s work in CEs began during the 1968 war-induced famine in Biafra in West Africa, during which staff documented the extent of severe malnutrition (*11*). CDC’s assessment of the health effects of the 1970 Bangladesh cyclone established epidemiologic approaches in humanitarian emergencies triggered by natural disasters (*12*). CDC published a compendium of disease control and public health surveillance programs used among Khmer refugees from Kampuchea (Cambodia) in Thailand during 1979–1980 (*13*), followed by a synthesis of accumulated knowledge about public health issues in CEs (*14*). In 1990, Toole and Waldman, among the first CDC staff dedicated to studying the epidemiology of CEs, published a paper on mortality rates among displaced populations, which established the use of a crude mortality rate (CMR) threshold to quantitatively define CEs (*15*). In 1994, CDC staff, as part of the Goma Epidemiology Group, conducted rapid cluster sample population surveys to document the unprecedented mortality rate among Rwanda refugees in Goma, Zaire (now Democratic Republic of the Congo) (*6*,*8*). After a systematic review of nutritional surveys in Somalia during the 1991–1992 famine, CDC staff provided recommendations for standardizing nutritional assessments in CEs (*16*). Toole et al. published on measles control in refugee settings in 1989 with special attention to how measles prevention policies during CEs differ from measles control in standard settings (*17*,*18*). In the 1990s and 2000s, CDC staff emphasized the burden of chronic diseases in CEs (*19*) and documented adverse mental health outcomes and social functioning among refugee and CE-affected populations (*20*–*23*) and, later, among national and international aid workers (*24*–*26*). In all activities, CDC worked to address the unique characteristics of humanitarian emergencies through development of epidemiologic methods, strengthening local capacity, improvement of surveillance, and evaluation of interventions.

CDC continues its work on humanitarian emergencies through its humanitarian emergency response branch, the Emergency Response and Recovery Branch (ERRB), in the Division of Global Health Protection, Center for Global Health. ERRB includes the Global Disease Detection Operations Center, the Global Rapid Response Team, the Global Response Preparedness Team, the Global WASH Team, and the Humanitarian Health Team, thus unifying CDC’s humanitarian emergency preparedness, alert, and response activities into a single program. Staff members work with the international humanitarian community to apply public health and epidemiologic science, develop tools and methods to understand health needs, and build the capacity and resilience of public health systems within these fragile settings. This article focuses on ERRB’s work in humanitarian emergency response over the past decade; its collaboration with response partners; the broad lessons that can be drawn from its work; and how it and other humanitarian health responders are adapting to address new threats to global health security, new needs of populations affected by CEs, and humanitarian emergencies at large.

## Response Descriptions

We retrospectively examined ERRB responses during 2007‒2016. To compile the responses that occurred during 2007‒2014, we abstracted data from past branch activities databases and publications. To ensure the completeness of this dataset, we compared it with a previously compiled comprehensive database of all emergencies worldwide, including CEs and natural disasters, for the same period (A. Culver, Emory University, pers. comm., 2015 Aug 4). Sources of CEs included in this database were the Center for Research on the Epidemiology of Disasters Complex Emergency Database (http://cedat.be) and the United Nations Office for the Coordination of Humanitarian Affairs Central Emergency Response Fund archives of funded responses (http://www.unocha.org/cerf/cerf-worldwide/allocations-country/2006-2017-country). Included events were those that affected >10,000 persons and had a documented CMR of >1 death/10,000 persons/day. Sources for natural disasters were the Center for Research on the Epidemiology of Disasters international disaster database (http://www.emdat.be) and Central Emergency Response Fund archives; inclusion required that the event affected >10,000 persons. To compile emergency responses for 2015 and 2016, we abstracted data from branch administrative and travel records. Responses were selected to reflect >1 response/year and to include those in which branch staff had a major role.

Of 14 selected emergencies over the past 10 years, nearly two thirds were CEs; the rest were natural and human-made disasters (Technical Appendix Table). Responses were conducted in Africa, Asia, Latin America and the Caribbean, Europe, and the Middle East; activities included providing technical assistance or directly conducting assessments and investigations, implementing and evaluating surveillance systems, developing guidelines, providing trainings, and coordinating interventions. All responses featured extensive collaboration with a variety of partners, including the US government, UN agencies, governmental health entities, and both national and international nongovernmental organizations (NGOs). To illustrate the breadth and impact of the CDC and its partners’ work in humanitarian emergency responses, we highlight activities performed in 3 cases (Technical Appendix Table).

## Case Studies

### Haiti Earthquake Response, 2010

On January 12, 2010, a 7.0 magnitude earthquake struck central Haiti, killing >200,000 persons and injuring another 300,000. The quake also created 1 million IDPs and massively disrupted public health and other basic services within an already fragile state. ERRB staff worked with the Pan American Health Agency and the Haiti Ministry of Public Health and Population to establish sentinel site surveillance for epidemic-prone infectious diseases at 51 health facilities across the country and in IDP camp clinics; these systems were instrumental in detecting the cholera outbreak that began in October 2010 (*27*,*28*). Recognizing that population displacement could exacerbate Haiti’s already poor access to improved water sources and sanitation facilities, ERRB staff and the Haiti National Directorate for Potable Water and Sanitation performed a rapid assessment of access to WASH services in 308 IDP settlements in February 2010 and found that <10% of sites met the minimum Sphere Project standards for emergency sanitation (<50 persons/latrine) (*29*). This work provided the impetus for the Haiti National Directorate for Potable Water and Sanitation and the humanitarian WASH sector to increase emphasis on improving WASH in IDP sites, actions that likely reduced the number of cases among IDPs early in the cholera epidemic (*30*,*31*).

The cholera epidemic was also the basis for a series of ERRB activities focused on improving access to clean water and proper sanitation in Haiti. Although WASH had been a core sector within ERRB, this epidemic led to an increased CDC emphasis on implementing and evaluating WASH interventions in CEs. In light of the ongoing burden of thousands of cholera cases in Haiti annually, WASH activities in Haiti are now a focus of health systems recovery work of ERRB and the CDC Haiti country office.

### Horn of Africa Famine and Displacement Response, 2011–2014

In 2011, a drought in the Horn of Africa led to severe food insecurity for 13 million persons, contributing to 30% acute malnutrition rates and declaration of famine in 3 regions of Somalia (*32*). Nearly 1 million Somali refugees fled to camps in Kenya and Ethiopia, and an additional 1.5 million persons were internally displaced within Somalia. Host populations in Kenya also experienced emergency rates of >25% acute malnutrition, and outbreaks of measles and cholera occurred.

In response, ERRB staff worked with the UN High Commissioner for Refugees (UNHCR) to strengthen its Health Information System (HIS) disease surveillance (http://www.unhcr.org/en-us/protection/health/4a3374408/health-information-system-toolkit.html), which led to identification of measles outbreaks in 2 refugee camps. Staff review of demographic profiles of outbreak cases led to an expansion of the target age group for vaccination from 6 months–14 years of age to 6 months–30 years of age (*33*,*34*). In a retrospective survey of deaths among 753 refugee families arriving at Dadaab, Kenya, ERRB staff and partners noted a doubling of CMR among refugees in transit (CMR 1.94, 95% CI 0.50–3.37) compared with that before departure (CMR 0.86, 95% CI 0.57–1.15), leading to aid agencies intervening during refugees’ journeys (*35*). ERRB’s evaluation of a blanket supplementary feeding program in northern Kenya, conducted with several collaborators, pointed out the need for more regular distribution of rations and strengthened interventions for acutely malnourished children (*36*). ERRB staff and UN partners reviewed and validated all aid groups’ nutrition and mortality surveys conducted in Somalia to ascertain the severity of the famine in some affected areas, thus directing aid (*32*). Until 2014, ERRB supported the Somalia communicable disease reporting surveillance system, designed to optimize early warning of outbreaks, by providing analysis and training; this system identified an outbreak of polio in 2013, enabling swift intervention (*37*).

For ERRB, the response to the Horn of Africa famine and displacement indicated the value of enhancing public health information quality, thereby guiding the allocation of humanitarian resources. ERRB’s response to this emergency also sharpened CDC’s capacity to respond to protracted emergencies over the course of several years, adapting responses to the changing public health needs across several sites simultaneously within a destabilized region. In addition, this response represented one of the first instances of ERRB’s providing remote support and monitoring of emergency public health activities.

### Syria Displacement Response, 2012‒Present

Antigovernment protests in Syria in 2011 devolved into an ongoing, multisided armed conflict that has devastated a previously middle-income country and destabilized the region. The UN Office for the Coordination of Humanitarian Affairs estimated, as of October 2016, that 13.5 million persons across the region were in need of humanitarian assistance. The war has caused the displacement of 4.8 million persons outside the country and 6.1 million within, totaling more than half of Syria’s population (*38*). The displacement crisis has strained resources in neighboring countries and beyond.

As in other protracted emergencies, ERRB’s work has spanned several years and multiple sectors. Branch staff helped UNHCR implement HIS for disease surveillance in Za’atari refugee camp in Jordan, including introduction of an outbreak response protocol. Thereafter, when HISshowed a decline in child vaccination rates in the camp area from 90% to 50%, aid partners conducted a measles vaccination campaign of 660,000 children. Working with the US Agency for International Development’s Office of Foreign Disaster Assistance and the Assistance Coordination Unit, staff also established and trained local users on the Early Warning Alert and Response Network in northern Syria, playing a fundamental role in establishing disease surveillance in non–government-controlled areas and increasing local public health capacity. This system detected a polio outbreak in 2013, initiating a vaccination campaign, and provided information on suspected cholera cases and measles and typhoid fever outbreaks. ERRB assisted UNHCR, UNICEF, and other partners in conducting cross-sectional representative cluster surveys of nutritional status of refugee children and women of reproductive age, finding a high prevalence of anemia in both groups and providing evidence to support a micronutrient fortification food program for refugees (*39*). ERRB and multiple collaborators performed an assessment of the Minimum Initial Services Package for reproductive health among the refugees from Syria residing in Jordan and instituted a protocol for clinical management of survivors of sexual violence after noting a lack of such services (*40*).

This response in Syria indicated the importance for the emergency health response community to support public health guideline and strategy development and program implementation across regional public health systems. The Syria displacement crisis also pointed out the need to develop responses for emergencies in middle-income regions of the world, where demographics, disease burden, and functionality of public health systems are different from those of sites of historic CEs.

## Discussion

Reflecting on these 3 case studies and the other listed ERRB humanitarian emergency responses, several overarching lessons for effective public health humanitarian emergency response emerge. First, because humanitarian emergency response requires engaging in a broad range of public health work within resource-limited, fragile, or insecure environments, successful response requires developing close working relationships with other humanitarian response organizations. For CDC, these partnering organizations include national governments; ministries of health; US government agencies (especially the Agency for International Development’s Office of Foreign Disaster Assistance and the Department of State Bureau of Populations, Refugees, and Migration); UN agencies, including the World Health Organization, UNHCR, and UNICEF; and national and international NGOs. At a basic level, these close relationships allow ERRB and other humanitarian responders access to CE settings. These collaborations encourage standardization of approaches across the international humanitarian emergency response community (*29*) and improved coordination of response (*6*,*18*). The common use of these standardized practices has been facilitated by the dissemination of the epidemiologic approaches and methods championed by CDC during humanitarian emergency responses and through CDC-trained staff going on to senior positions at UN agencies. Finally, these collaborations permit ERRB and similar organizations to provide technical assistance while partners such as national ministries of health, UN agencies, and NGOs take the lead in implementation of interventions.

Second, because public health emergency responses often take place within the context of mass population displacement and fragile states, CDC and other responders must develop and apply epidemiologic methods and tools to be used in challenging and resource-limited settings. ERRB has contributed to several such tools. In the nutrition sector, ERRB enhanced the application of the emergency nutrition assessment software that facilitates survey planning, data collection, and analysis of anthropometric indices (http://smartmethodology.org/survey-planning-tools/smart-emergency-nutrition-assessment) and led the technical development of the Community-based Management of Acute Malnutrition report for monitoring programs to manage malnutrition in emergencies (*41*). In the communicable diseases sector, ERRB helped develop the evaluation tool for tuberculosis in resource-limited, refugee, and postconflict settings (https://www.cdc.gov/globalhealth/healthprotection/errb/researchandsurvey/tbtool.htm). In the sexual and gender-based violence sector, branch staff contributed to the guidelines for integrating gender-based violence interventions in humanitarian action (http://gbvguidelines.org/wp/wp-content/uploads/2015/09/2015-IASC-Gender-based-Violence-Guidelines_lo-res.pdf). Across these and other sectors, in settings where the evidence base for interventions is limited, CDC focuses on strengthening the accuracy of data to build a solid evidence base for interventions to guide humanitarian response, enhance global health security, and prevent morbidity and mortality.

Third, effective emergency responses must adapt to changing needs of emergency-affected populations. Humanitarian emergencies, especially CEs, which exacerbate the fragility of politically weak and unstable regions, could last several years without a clear endpoint. Although dramatically elevated mortality rates might decrease as a CE moves from an acute emergency to a postemergency phase, populations continue to be vulnerable to many of the same health risks. As the humanitarian response evolves and becomes better established, responders might need to strengthen disease surveillance, review and interpret public health data, and improve capacity of local or national public health systems. Responders must maintain a commitment to improving the function and resilience of public health systems within these fragile settings.

Fourth, ERRB’s work supports the work of CDC to prevent, detect, and respond to public health threats in fragile states under conditions that can result in regionally destabilizing effects and threaten global health security. Responding effectively requires that ERRB and other responders recognize 3 global patterns in population displacement: urbanization of the displaced, a shifting disease burden that includes noncommunicable diseases, and increasing security restrictions in areas of displacement. Understanding the unique aspects of urbanization of the displaced, moving away from the rural camp–based models of the past, suggests the need to change epidemiologic methods of surveillance and population assessment. In addition, because the displaced are increasingly likely to need assistance for noncommunicable, chronic diseases and access to long-term health services, compared with displaced populations in the past (*9*), the humanitarian emergency response community’s areas of expertise must expand to include this sector. Increasing security restrictions have sometimes prevented, and will likely continue to prevent, CDC staff and the humanitarian community from physically accessing certain displaced populations. Furthermore, CDC is the public health agency of the US government and not a humanitarian agency, and thus, ERRB’s responses are limited in ways that those of humanitarian agencies are not. These limitations include where, under what circumstances, and with which partners CDC staff can work. To address these limitations, ERRB staff is working to formalize remote support and program evaluation without sacrificing quality or comprehensiveness of assistance. More broadly, however, ERRB relies on humanitarian agencies to continue using epidemiologically sound public health approaches to guide evidence-based, effective interventions when CDC is precluded from responding.

Finally, ERRB responses show that response expertise is most useful when deployed early in an emergency and with a sustained presence. To that end, ERRB’s Global Rapid Response Team quickly matches needs in the field with expertise available from within ERRB and across the entire CDC, and these responders can provide longer-term support. In its first year of existence, the Global Rapid Response Team deployed >200 staff members to various emergency responses, including for Hurricane Matthew in Haiti in October 2016.

As the numbers affected by and intensity of humanitarian emergencies increase, ERRB and other response organizations must provide broader assistance. To that end, ERRB collaborates with partners; contributes to epidemiologic tools to be used in humanitarian emergencies; and, through the Global Rapid Response Team, responds more quickly and with more staff. The next steps for ERRB and other responders include improving capacity and resilience of public health systems in fragile states; understanding the public health implications of long-term, urban-based displacements; adding a focus on noncommunicable diseases; and providing remote epidemiologic support in a systematic way. In settings where ERRB staff, as representatives of a US government agency, cannot respond, CDC’s evidence-based interventions for emergencies are still implemented because of branch efforts in building local capacity for emergency response and training public health practitioners who then move on to work with humanitarian agencies. In these ways, ERRB continues to apply public health science to save lives in humanitarian settings while also working on the forefront of response-purposed detection and preparing a global health response workforce.

Technical Appendix Select Centers for Disease Control and Prevention Emergency Response and Recovery Branch humanitarian emergency responses conducted, by year, type of emergency, activity, and partner, 2007–2016.
