# Supplementary material for: Centers for Disease Control and Prevention Public Health Response to Humanitarian Emergencies, 2007–2016
Source: Emerg Infect Dis. 2017 Dec;23(Suppl 1):S196–202. doi: 10.3201/eid2313.170473 (PMC5711329; doi:10.3201/eid2313.170473)
Supplement: Technical Appendix — Select Centers for Disease Control and Prevention Emergency Response and Recovery Branch humanitarian emergency responses conducted, by year, type of emergency, activity, and partner, 2007–2016. [file 17-0473-Techapp-s1.pdf]

Article DOI: https://doi.org/10.3201/eid2313.170473

Centers for Disease Control and Prevention Public Health Response to Humanitarian Emergencies, 2007–2016

Technical Appendix

Technical Appendix Table. Select Centers for Disease Control and Prevention Emergency Response and Recovery Branch humanitarian emergency responses conducted, by year, type of emergency, activity, and partner, 2007–2016*

Year	Type	Country or region, nature of emergency	Description of activities	Partners for activities
2007	CE	Sudan (Darfur, southern Sudan) displacement crises of refugees and IDPs	1. Supported USAID's OFDA DART<br>2. Conducted emergency food security and nutrition assessment<br>3. Assessed clinical care access among returning refugees<br>4. Provided technical assistance for WASH interventions for hepatitis E outbreak	1. USAID/OFDA<br>2. World Food Programme, UNICEF<br>3. Southern Sudan Ministry of Health, UNHCR<br>4. Southern Sudan Ministry of Health, WHO
2008	CE	Zimbabwe political instability and cholera outbreak	1. Supported DART on WASH activities for cholera response<br>2. Evaluated cholera WASH response activities<br>3. Investigation of high rates of community-based cholera deaths (*1*)	1. USAID/OFDA<br>2. UNICEF<br>3. Zimbabwe Ministry of Health and Child Welfare, UNICEF, Medical Emergency Relief International, WHO
2009	CE	Sri Lanka displacement crisis	1. Conducted community and household assessment of vaccination rates, healthcare access, death, prevalence of injury, disability, mental health conditions, and sexual and gender-based violence (*2*)<br>2. Assessed mental health of national humanitarian aid staff (*3*)	1. UNICEF, Sri Lanka Ministry of Health<br>2. Consortium of Humanitarian Agencies, Antares Foundation
2010	ND and CE	Haiti earthquake and cholera outbreak	1. Established sentinel site surveillance systems (*4,5*)<br>2. Conducted nutrition assessment and provided technical assistance for supplemental immunization activities<br>3. Conducted rapid assessments of water access and quality in IDP camps and cholera-affected areas<br>4. Provided technical assistance with maternal surveillance<br>5. Supported UN coordination of response	1. PAHO, Haiti MSPP<br>2. UNICEF, MSPP<br>3. Haiti DINEPA<br>4. UNFPA, PAHO<br>5. PAHO, MSPP
2010	ND	Pakistan floods	1. Expanded and strengthened EWARN (*6*)<br>2. Conducted emergency nutrition surveys in flood-affected areas<br>3. Supported WHO in coordination of flood response	1. Pakistan National Institute of Health, National Emergency Preparedness and Response Network, WHO<br>2. UNICEF<br>3. WHO, Pakistan Ministry of Health

Year	Type	Country or region, nature of emergency	Description of activities	Partners for activities
2011–2014	CE	Horn of Africa famine and displacement crisis	1. Strengthened UNHCR Health Information System surveillance<br>2. Strengthened WASH preparedness in refugee camps, trained healthcare workers on cholera management<br>3. Described measles outbreaks in 2 refugee camps in Kenya and Ethiopia (*7*)<br>4. Assisted in coordination of measles vaccination campaign in Somalia<br>5. Investigated mortality rates among refugees (*8*)<br>6. Conducted prospective cohort study to assess impact of blanket supplementary feeding in nutritional emergencies (*9*)<br>7. Conducted expert review of nutrition quality and mortality data from affected areas in Somalia for possible famine declaration (*10*)<br>8. Identified gaps in emergency obstetric care<br>9. Planned and implemented measles vaccination campaign in Somalia<br>10. Supported Health Coordinator in refugee camp in Ethiopia<br>11. Supported OFDA DART<br>12. Supported Somali communicable disease reporting system improvement (*11*)	1–3. UNHCR<br>4. UNICEF<br>5. UNHCR, Kenya Medical Research Institute, Food Security and Nutrition Analysis Unit Somalia<br>6. World Food Programme, UNICEF, Arid Lands Development Focus Kenya, International Rescue Committee, Islamic Relief, Merlin, Oxfam, Save the Children, World Vision<br>7. FAO/Food Security and Nutrition Analysis Unit Somalia<br>8. UNFPA<br>9. UNICEF<br>10. UNHCR, Ethiopia Ministry of Health<br>11. USAID/OFDA<br>12. WHO Somalia
2012	Human-made disaster	Republic of Congo munitions explosion	1. Established EWARN	1. Republic of Congo Ministry of Health, WHO
2012–2016	CE	South Sudan displacement crises of refugees and IDPs	1. Evaluated EWARN<br>2. Conducted hepatitis E outbreak investigation and response<br>3. Conducted nodding syndrome investigation<br>4. Assessed mortality rate among refugees<br>5. Initiated community-based pregnancy surveillance<br>6. Provided technical assistance for managing acute malnutrition programs in emergencies, reviewed and validated all nutrition and mortality surveys (*12*)	1. WHO, South Sudan Ministry of Health<br>2. UNHCR, Medecins Sans Frontieres<br>3. Ministry of Health, South Sudan<br>4. UNHCR<br>5. International Rescue Committee<br>6. Ministry of Health, South Sudan, UNICEF
2012–2016	CE	Syria displacement crisis	1. Implemented Health Information Systems in Jordan refugee camp<br>2. Coordinated mass measles vaccination campaign in Jordan<br>3. Trained staff, implemented EWARN in northern Syria with subsequent evaluation<br>4. Conducted EWARN training in Jordan for EWARN surveillance officers in Syria<br>5. Conducted training on infant and young child feeding in Jordan<br>6. Developed and evaluated tuberculosis screening and treatment strategy among refugees in Jordan<br>7. Assisted in evaluating mental health programs among refugees in Jordan (*13*)<br>8. Conducted nutrition surveys in Syrian refugee camps and among Syrian refugees living in host community in Jordan (*14*)<br>9. Assisted in planning, design, and implementation of nutrition surveys in opposition-controlled areas of northern Syria<br>10. Supported OFDA Response Management Team in Washington and the DART on polio response in Jordan<br>11. Supported oral polio vaccination campaign in northern Syria from Turkey<br>12. Provided infant and young child feeding support to refugees in Croatia<br>13. Evaluated minimum initial services package for reproductive health in refugee sites in Jordan (*15*)<br>14. Performed rapid health needs assessments in Syrian refugee camps in Greece	1. UNHCR<br>2. UNICEF<br>3. USAID/OFDA, Assistance Coordination Unit<br>4. WHO, Assistance Coordination Unit<br>5. UNICEF<br>6. UNHCR, Jordan Ministry of Health, International Organization for Migration, WHO<br>7. International Medical Corps<br>8. UNICEF, UNHCR<br>9. UNICEF<br>10. USAID/OFDA<br>11. UNICEF<br>12. UNICEF<br>13. UNHCR, Women's Refugee Commission, Boston University School of Public Health, UNFPA, International Rescue Committee<br>14. Department of State US Embassy, Government of Greece

Year	Type	Country or region, nature of emergency	Description of activities	Partners for activities
2013	ND	Philippines typhoon	1. Strengthened EWARN<br>2. Contributed to restoration of vaccine services, assisted with measles vaccination campaign<br>3. Developed cholera preparedness plan	1. WHO, Philippines Department of Health<br>2. UNICEF<br>3. UNICEF, Philippines Department of Health
2014	CE	Ukraine displacement crisis	1. Coordinated nutrition sector humanitarian response<br>2. Conducted health and nutritional assessment of older adults residing in conflict areas<br>3. Assessed infant and young child feeding practices among displaced population<br>4. Established and evaluated anemia surveillance	1–4. UNICEF, Ukraine Ministry of Health
2014–2016	CE	Iraq displacement crisis	1. Analyzed injury surveillance data (*16*)<br>2. Provided technical assistance to support WASH and outbreak control response to cholera outbreak<br>3. Provided technical assistance to implement and evaluate oral cholera vaccination campaign (*17*)<br>4. Evaluated EWARN	1. WHO, Iraq Ministry of Health<br>2. USAID/OFDA, UNICEF, Iraq Ministry of Health<br>3. WHO, Iraq Ministry of Health and Sanitation, UNICEF, Iraq Red Crescent Society<br>4. WHO, Iraq Ministry of Health and Sanitation
2016	CE	Nigeria displacement crisis	1. Conducted survey to estimate acute malnutrition and infectious diseases rates<br>2. Conducted expert review on nutrition quality and mortality rate data in affected areas for possible famine declaration	1. UNICEF<br>2. FAO
2016	ND	Haiti hurricane	1. Evaluated cholera treatment protocol and surveillance system<br>2. Provided technical assistance to improve WASH interventions for cholera prevention<br>3. Assessed access to HIV and tuberculosis clinical care	1. Haiti MSPP<br>2. USAID/OFDA, DINEPA, International Organization for Migration<br>3. MSPP

*CE, complex emergency; DART, Disaster Assistance Response Team; DINEPA, National Directorate for Potable Water and Sanitation; EWARN, Early Warning Alert and Response Network; FOA, Food and Agriculture Organization; IDP, internally displaced person; ND, natural disaster; MSPP, Ministry of Public Health and Population; OFDA, Office of Foreign Disaster Assistance; PAHO, Pan-American Health Agency; UNHCR, United Nations High Commissioner for Refugees; UNICEF, United Nations Children's Fund; UNFPA, United Nations Population Fund; USAID, US Agency for International Development; WASH, water, sanitation, and hygiene; WHO, World Health Organization.

References

1. Morof D, Cookson ST, Laver S, Chirundu D, Desai S, Mathenge P, et al. Community mortality from cholera: urban and rural districts in Zimbabwe. Am J Trop Med Hyg. 2013;88:645–50. PubMed http://dx.doi.org/10.4269/ajtmh.11-0696

2. Husain F, Anderson M, Lopes Cardozo B, Becknell K, Blanton C, Araki D, et al. Prevalence of war-related mental health conditions and association with displacement status in postwar Jaffna District, Sri Lanka. JAMA. 2011;306:522–31. PubMed http://dx.doi.org/10.1001/jama.2011.1052

3. Lopes Cardozo B, Sivilli TI, Crawford C, Scholte WF, Petit P, Ghitis F, et al. Factors affecting mental health of local staff working in the Vanni Region, Sri Lanka. Psychol Trauma. 2013;5:581–90. PubMed http://dx.doi.org/10.1037/a0030969

4. Centers for Disease Control and Prevention. Launching a national surveillance system after an earthquake—Haiti, 2010. [Erratum in MMWR Morb Mortal Wkly Rep. 2010;59:993.] MMWR Morb Mortal Wkly Rep. 2010;59:933–8. PubMed

5. Centers for Disease Control and Prevention. Rapid establishment of an internally displaced persons disease surveillance system after an earthquake—Haiti, 2010. MMWR Morb Mortal Wkly Rep. 2010;59:939–45. PubMed

6. Centers for Disease Control and Prevention. Early warning disease surveillance after a flood emergency–Pakistan, 2010. MMWR Morb Mortal Wkly Rep. 2012;61:1002–7. PubMed

7. Navarro-Colorado C, Mahamud A, Burton A, Haskew C, Maina GK, Wagacha JB, et al. Measles outbreak response among adolescent and adult Somali refugees displaced by famine in Kenya and Ethiopia, 2011. J Infect Dis. 2014;210:1863–70. PubMed http://dx.doi.org/10.1093/infdis/jiu395

8. Centers for Disease Control and Prevention. Notes from the field: mortality among refugees fleeing Somalia—Dadaab refugee camps, Kenya, July-August 2011. MMWR Morb Mortal Wkly Rep. 2011;60:1133. PubMed

9. Shahpar C, Talley L. Impact evaluation of BSFP during a nutrition emergency in Kenya. Field Exch. 2014;47:68.

10. Centers for Disease Control and Prevention. Notes from the field: malnutrition and mortality—southern Somalia, July 2011. MMWR Morb Mortal Wkly Rep. 2011;60:1026–7. PubMed

11. Cookson STAA, Everard M, Popal GR, Clarke KR, Husain F. Success with disease surveillance in Somalia. BMJ. 2013;347:21.

12. Michalska A, Leidman E, Fuhrman S, Mwirigi L, Bilukha O, Basquin C. Nutrition surveillance in emergency contexts: South Sudan case study. Field Exch. 2015;50:73. PubMed

13. International Medical Corps. Evaluating mental health case management services for refugees in Jordan. 2015 [cited 2016 Oct 30]. https://www.state.gov/documents/organization/250834.pdf

14. Bilukha OO, Jayasekaran D, Burton A, Faender G, King'ori J, Amiri M, et al.; Division of Global Health Protection, Center for Global Health, CDC; Centers for Disease Control and Prevention. Nutritional status of women and child refugees from Syria – Jordan, April-May 2014. MMWR Morb Mortal Wkly Rep. 2014;63:638–9. PubMed

15. Krause S, Williams H, Onyango MA, Sami S, Doedens W, Giga N, et al. Reproductive health services for Syrian refugees in Zaatri Camp and Irbid City, Hashemite Kingdom of Jordan: an evaluation of the Minimum Initial Services Package. Confl Health. 2015;9(Suppl 1):S4. PubMed http://dx.doi.org/10.1186/1752-1505-9-S1-S4

16. Leidman E, Maliniak M, Sultan AS, Hassan A, Hussain SJ, Bilukha OO. Road traffic fatalities in selected governorates of Iraq from 2010 to 2013: prospective surveillance. Confl Health. 2016;10:2. PubMed http://dx.doi.org/10.1186/s13031-016-0070-0

17. Lam E, Al-Tamimi W, Russell SP, Butt MO, Blanton C, Musani AS, et al. Oral cholera vaccine coverage during an outbreak and humanitarian crisis, Iraq, 2015. Emerg Infect Dis. 2017;23:38–45. PubMed http://dx.doi.org/10.3201/eid2301.160881
